# How Can Jewish and Non-Jewish People Collaborate to Improve Healthcare in the US? Considering Community, Autonomy, and Solidarity

**DOI:** 10.5041/RMMJ.10502

**Published:** 2023-07-31

**Authors:** Zackary Berger

**Affiliations:** 1 Johns Hopkins School of Medicine, Baltimore, MD, USA; 2 Johns Hopkins Bloomberg School of Public Health, Baltimore, MD, USA; 3 Johns Hopkins Berman Institute of Bioethics, Baltimore, MD, USA

**Keywords:** Anti-Semitism, bioethics, community, history, Judaism, US healthcare

## Abstract

The coronavirus 2019 (COVID) pandemic has highlighted the ways in which municipal, state, and Federal agencies in the USA have failed to address the inequities of present-day health systems. As alternative organizing centers outside these agencies, local communities are potentially situated to redress the inequities of present-day health systems in a collaborative manner that demonstrates solidarity by supplementing a purely scientific model of medicine and healthcare. In the mid-twentieth century, the Black Panthers, a revolutionary African American nationalist organization that focused on socialism and self-defense, introduced highly influential free clinics, which sought to bring expertise to the Black community on their own terms. This required bringing the benefits of biomedicine to those who customarily had not seen them. By extension, their approach raises questions regarding community- and expertise-centered approaches for the Jewish community: how is it engaged in healthcare for itself (in its diverse subcategories) and for others? Moreover, understanding how the Jewish community has been ill-served by present-day health-care systems might spur Jewish institutions to reimagine how healthcare should work.

## INTRODUCTION

While there exists a robust literature on individual obligations in Jewish bioethics, there is little discussion in the ethical literature on the responsibility of Jewish institutions, communal or otherwise, regarding the health of non-Jews, or how Jews and non-Jews should collaborate in improving the health of the community. Whether Jews should consider the health and well-being of non-Jews as important is outside the scope of this paper; nevertheless, a humanistic notion of the worth of all human beings, or the theological concepts of humankind being created in God’s image, can be cited in this regard.^1^ If one is of the opinion that non-Jews are not the proper topic of consideration of Jewish communal institutions, the fact that Jews most often, historically, lived as neighbors to non-Jews should be noted.^2^ Furthermore, the relationships between Jews and non-Jews have been a topic of much discussion throughout the development of Jewish religious, legal, and ethical literature.^3^ This paper takes the assumption that Jewish people cannot avoid some involvement with their non-Jewish neighbors, and, whether for better or for worse, that involvement should be considered normatively. During the COVID pandemic, governmental public health agencies in the USA and elsewhere have failed to ensure equitable treatment, testing, support, or mitigation of the pandemic, especially among disadvantaged groups and ethnic groups characterized by mistrustful or conflict-ridden relationships with those agencies. Exclusively biomedical approaches to the pandemic, according to which technical developments in testing, treatment, and vaccination would end the pandemic, have proven insufficient.

Community-centered models of care emphasize interaction with expertise according to the needs of that specific community at which the care is directed. These models have had multiple sources, but one influential implementation was in the Free Clinics established by the Black Panther Party in the US of the late twentieth century.^4^ These free clinics sought to engage the community in questions of prioritization of healthcare. What did African Americans most need to ensure their health? What needs from Black Americans were overlooked by the health-care establishment, and what innovations in care delivery, collaboration with communities, testing, and treatment were required to address those unmet needs?^5^ Every community can ask those questions of the health-care system as it currently stands, and the Jewish communities in their various kinds in the US and elsewhere can, in turn, ask whether governmental public health agencies are meeting their particular needs. As sources of power in their relationship with the state, Jewish institutions might ask how healthcare can be envisioned in terms appropriate for US Jews. In understanding such institutions’ particular needs, a fine-grained understanding of culturally appropriate health-care delivery to US Jews should be achieved. There is a growing literature of such appreciation in the case of US Charedi Jews, for instance.^6^

For their part, African American, Latino, and Native American organizations have played central roles in highlighting the inequitable aspects of the US response to COVID in Federal, state, and urban jurisdictions. African American organizations have made testing and vaccinations available, as well as providing social support which Federal and local jurisdictions have not provided. Latino organizations have focused on the needs of undocumented immigrants, a group not equitably served by the Federal government. With their own diverse needs, Native Americans have nonetheless been vaccinated at rates comparable to other groups largely through the work of community organizations collaborating with the Indian Health Service.^7^

At the same time, the COVID pandemic has had severe effects among Jewish communities in the US, Israel, and elsewhere, particularly among the Charedim.^8^ If the Federal and state agencies have not equitably implemented a pandemic response, is there a way in which different communities can work together to make all US residents the beneficiaries of effective public health interventions? Jewish organizations can also answer this question. What kind of relationship should there be between the Jewish and non-Jewish communities? How should the Jewish community govern its own healthcare, and what are its obligations to the surrounding communities? In order to give a range of possible answers to such questions, there needs to be a serious consideration of the history of the US Jewish community and how the current relationship of US Jews to Jewish and non-Jewish health institutions came to their current status and role in the US.

A relevant example in this regard can be found in another influential implementation of community care and free clinics, on the part of Jack Geiger and other US progressive doctors, not coincidentally Jewish people.^9^ There are at least three domains in which these relationships between US Jews and health institutions are relevant: (1) consideration of the disproportionate representation of Jewish people among health-care providers and leaders;^10^ (2) the influence that Jewish groups have had in the ownership and placement of health-care institutions;^10^ and (3) how the Jews as a minority community have been affected by at least some of the same health-care inequities that have affected other communities, as well as their being victimized by hate and violence.^9^

How has the involvement of US Jewish people in the US health-care system changed, and how has that affected the US health-care system as it serves non-Jewish people? What can we learn about the ways Jewish and non-Jewish people and organizations have collaborated in the health-care system, and is that knowledge applicable to our present situation?

## HISTORICAL OVERVIEW OF JEWISH HOSPITALS AND HEALTHCARE IN THE USA

The modern Jewish hospital in the US, dating from the nineteenth century, historically served Jews and non-Jews, such as the first Jewish hospital established in Cincinnati, Ohio, in 1850.^11^ The New York Jewish Hospital, which later became The Mount Sinai Hospital, also served both communities.^11^ In particular, these hospitals acknowledged their shared responsibility for the care of the indigent from all communities. Their founding was also a reaction to the anti-Semitism of the time, which denied Jewish doctors and other professionals an equal place in existing hospitals.^11^ Note that these hospitals, which served primarily Black and White patients, did not provide care that was more equitable or more progressive than other, non-Jewish hospitals of the day.

Thus, from the very start of the establishment of Jewish hospitals in the US, Jewish–non-Jewish interactions in training, administration, patient care, and financing were part of standard hospital operations.

In the early twentieth century, Jewish and Irish immigrants were heavily represented in US cities and among the patients cared for by Jewish hospitals. In the 1910s and 1920s, the Great Migration of African Americans from the Southern to the Northern US was met with racist violence in Northeastern cities—and also increased the proportion of non-Jewish patients cared for by these Jewish hospitals. Jewish fraternal and communal organizations continued to support the hospitals which tended to their members. The Jewish population began to migrate to the suburbs; however, their hospitals stayed behind, taking care of some immigrants but also segregated and racialized African Americans.

After World War II, US hospitals became centers of experimental research and associated therapies.^12^ American medical institutions began to recruit research subjects from the surrounding, mostly Black, community.^13^ The same period saw an ever-tighter connection of American Jewish philanthropy with the capitalist state and liberal economy. Jewish philanthropies became mechanisms to accumulate tax-free capital. Hospitals became less attractive philanthropic investments, especially smaller hospitals which found it difficult to compete with larger hospital networks.^14^ Some Jewish hospitals still exist as exceptions to this phenomenon, and a history of these still-remaining Jewish hospitals remains to be written; a recent contribution to the topic attempts to address what makes contemporary Jewish hospitals different from non-Jewish hospitals.^15^

While the original Jewish hospitals served both the Jewish and non-Jewish communities, Jewish hospitals entered into the latter part of the twentieth century without explicitly distancing themselves from the segregated health-care system of the US; until the 1960s, separate hospitals existed for Black and White patients. The 1946 Hospital Funding and Construction Act, commonly known as the Hill–Burton Act, provided Federal funding for the construction of additional hospitals. Separate but equal (i.e. de-facto segregated) hospitals were allowed under the original legislation. It was only in 1964, via the Federal court Simkins v. Cone decision, that these provisions were overturned and such segregation forbidden under the Act.^16^ While the Civil Rights Act of 1964, soon thereafter, theoretically outlawed such de-facto segregation, it lives on in the two-tiered system of healthcare found for Black and White US residents.^17^

By the late twentieth century, community hospitals were confronted with new challenges. Many became part of larger for-profit hospital networks or academic medical centers. The latter tend to prioritize research funding instead of healthcare for the poorer urban residents living in close proximity to their facilities.^1^ Jewish hospitals, which are less competitive economically, have not necessarily played a leading role in these transformations, but were carried along with them.^18^ US Jewish organizations, particularly philanthropic ones, have played a significant role in shaping the internal framework of the US Jewish community, “where the meanings of American Jewish identity and social responsibility are negotiated, debated, and imposed.”^19^

## COVID, ANTI-SEMITISM, AND HEALTH

Since American Jewish hospitals have served non-Jews and continue to do so, the issue of anti-Semitism in the US raises important questions regarding how Jewish–non-Jewish collaborations should be managed within the American health-care system. Economic inequities underlay much of the differential burden of the COVID pandemic, and anti-Semitism has been found to be one of the responses to that pandemic.

Moshe Postone posited that contemporary anti-Semitism has its roots in a search for a single source for the ills born of capitalism.^20^ This theory covers some manifestations of contemporary anti-Semitism in the US, but not others. Different manifestations of anti-Semitism provide an image of what is broken in America’s current arrangements. Specifically, in right-wing circles, COVID has been blamed on Jewish people and is seen as (yet) another example of world domination by those with financial and political power.^8^ But another kind of anti-Semitism, both buttressed by and related to governmental control of Jews, should be highlighted as particularly relevant to the effect of the COVID pandemic on the lives of Charedi Jews. The directives of New York’s mayor Bill de Blasio and Governor Andrew Cuomo were specifically aimed at that community, including, in October, 2020, closures of religious schools and prohibitions on religious gatherings of more than 10 people.^21^ Moreover, De Blasio and Cuomo had inherited the inequitable practices that have historically differentially affected geographical divisions of New York City, including areas with a highly dense Charedi population.^22^

The American Jewish experience is not homogeneous, neither with regard to anti-Semitism nor during the COVID pandemic. Members of the Charedi community had various responses to Cuomo’s and De Blasio’s directives: some Charedim avoided that control; some welcomed it; some saw their fate as being bound up with their neighbors; and some saw themselves as a saving remnant. Yet others experienced COVID itself as a metaphysical or theurgic instance of social disintegration.^23^–^24^ Charedim were not the only ones targeted by state and city health-care agencies; other groups were, as well. In the 1970s, a Puerto Rican activist group, the Young Lords, carried out several direct actions and protests designed to call attention to the inequities of various New York governmental and public health systems, including hospitals and sanitation. In one well known incident, the Young Lords took over Lincoln Hospital in the Bronx, with Jewish physicians collaborating with them. Thus, systemic inequities were foundational to collaboration between ethnic groups.^25^

It should be mentioned that while the Charedim are sometimes understood as Jewish separatists par excellence, recent literature has pointed out that Charedi communities have interacted with non-Jews with political sophistication, forging alliances (without ignoring differences) for shared needs within US urban society, particularly in the Williamsburg neighborhood in Brooklyn, New York.^26^ In other words, rather than an example of Jewish isolation, Charedi society provides an example of collaboration and solidarity with other non-mainstream ethnic groups.

## HEALTH-CARE DISTRIBUTION AMONG US JEWS AND FUTURE SOLIDARITY

The US Jewish community today is characterized by wealth and inequality.^27^ While Jewish hospitals were initially founded to treat the indigent, Jewish hospitals are much fewer in number today and are by and large no longer independent institutions. Jewish people can access the most rarefied healthcare, while others lack the financial means to pay for their medical care.^28^ Both Charedim and non-Charedim (Conservative, Reform, Secular, etc.) experience inequities, mental and physical health challenges, and economic shocks in the wake of the COVID pandemic. All of them have experienced anti-Semitism, even though they live with and depend on non-Jewish people. However, these challenges point to possible opportunities for restructuring such institutions towards solidarity and equity in healthcare. In so doing, the linked histories of Jewish–non-Jewish roles in US healthcare would be recognized, and existing Jewish institutions would have the opportunity to realign in recognition of contemporary inequities. The American health-care system has negatively affected vaccination rates among the Charedim; such mistrust dates at least to the measles epidemic of 2019, if not before.^29^ However, the US Jewish sectors that have embraced and encouraged vaccination among the Jewish community—including the Charedim—do not appear to be facilitating such outreach for their affected non-Jewish neighbors. Which American Jewish federations are requesting funding from their member institutions to promote equity for the non-Jewish individuals who help keep the American Jewish institutions open?

A potential start to framing and addressing these questions could include establishing collaborative transparent relationships, so-called “networking solidarities,”^30^ that acknowledge the current differential access to power, resources, and expertise. Such solidarities would also recall the history of US Jewish healthcare and its service to, as well as interaction with, non-Jewish individuals and organizations.

## Abbreviations

COVID: coronavirus disease 2019
